# Heterogeneity among exercise participants: latent profile analysis and differential persistence pathways based on the pressure-support-motivation (PSM) framework

**DOI:** 10.3389/fpubh.2026.1890434

**Published:** 2026-07-24

**Authors:** Aojie Feng, Liwen Fan, Yuxuan Li, Zhifeng Zhao

**Affiliations:** School of Art, Soochow University, Suzhou, China

**Keywords:** appearance anxiety, exercise adherence, latent profile analysis, person-centered approach, PSM framework

## Abstract

**Background:**

Early dropout remains a major challenge in exercise adherence. Most existing studies adopt variable-centered approaches that overlook individual heterogeneity in psychological resources. This study aimed to identify distinct latent profiles of exercise participants and examine how the relationships between appearance anxiety, social support, and motivational resources with exercise persistence differ across profiles.

**Methods:**

A cross-sectional survey was conducted among 625 Chinese adults aged 18–45 who exercised at least once per week. Integrating Objectified Body Consciousness Theory, Social Cognitive Theory, and Self-Determination Theory, we proposed a Pressure-Support-Motivation (PSM) framework. Latent profile analysis (LPA) was performed using six indicators: appearance anxiety, social support, autonomous motivation, self-efficacy, body esteem, and hedonic motivation. Multi-group structural equation modeling (SEM) was subsequently used to compare path coefficients across profiles.

**Results:**

LPA identified four distinct profiles: Intrinsically Driven (28.3%), Anxiety-Transformed (24.2%), Socially Dependent (26.1%), and Motivation-Deficient (21.4%). Exercise persistence differed significantly across profiles, with the Intrinsically Driven group showing the highest levels and the Motivation-Deficient group the lowest. Multi-group SEM revealed differentiated mechanisms: body esteem and hedonic motivation showed stronger associations with persistence in the Intrinsically Driven profile; self-efficacy and autonomous motivation were most strongly linked in the Anxiety-Transformed profile, where appearance anxiety was positively associated with persistence; social support had the strongest direct association with persistence in the Socially Dependent profile; and most paths were weak or non-significant in the Motivation-Deficient profile.

**Conclusion:**

This study demonstrates substantial heterogeneity in the psychological drivers of exercise persistence. The findings provide preliminary profile-based evidence for the PSM framework and highlight the conditional role of appearance anxiety. Practically, the results support the development of tailored, profile-specific interventions and the design of adaptive digital health tools to improve long-term exercise adherence.

## 1 Introduction

Driven by visual culture and health awareness, exercise has become a core lifestyle (1). In China, physical activity participation and the fitness market are growing, yet the gap between exercise intention and actual behavior remains wide: most adults desire to exercise, but long-term adherence is low (2). Early dropout is common, highlighting the challenge of sustaining behavior. This raises key questions: Are exercisers a homogeneous group? Do adherence mechanisms differ?

Existing research spans health psychology (self-efficacy, goal setting; (3)), sociology (group norms, social capital; (4)), and consumer culture (body anxiety, appearance management; (5)). However, three limitations persist. First, variable-centered approaches overlook individual heterogeneity. Structural equation modeling and regression assume uniform causal mechanisms (6, 7), but social support may buffer stress for some and burden others. Second, theoretical integration is lacking. Appearance anxiety (8), social support (9), and self-determined motivation (10) are often studied separately; a unified framework is missing. Third, precision interventions lack grounding. Homogeneity-based strategies ignore individual differences. Moreover, appearance anxiety, pervasive in digital and visual cultures, is often seen as a negative stressor, yet many highly dissatisfied individuals maintain long-term exercise—a paradox requiring examination of boundary conditions.

To address these issues, we shift from a variable-centered to a person-centered perspective (11). Latent Profile Analysis (LPA) identifies distinct latent categories based on multidimensional response patterns (12). While person-centered methods have been used in exercise psychology (e.g., (13, 14)), they often focus on single dimensions, lacking integration of appearance anxiety, social support, and self-driven factors. Compared with variable-centered methods, latent profile analysis is more suitable for testing the heterogeneity hypothesis of this study. Regression or traditional SEM estimates population-averaged effects, implicitly assuming that different individuals follow the same parametric structure. However, under the PSM framework, pressure, support, and motivational resources may co-occur in different configurations, thereby forming multiple psychological profiles characterized by “the same variables, but different configurations.” In such cases, population-averaged effects may be “canceled out” (for instance, appearance anxiety may be positively associated with persistence in one subgroup, while being uncorrelated or negatively associated in another), thus obscuring key differences. LPA identifies latent subgroups at the level of joint distribution of multiple indicators, allowing this study to not only answer “which variables are associated with persistence,” but also to determine “which types of individuals are more likely to exhibit certain resource combinations in relation to persistence.”

This study integrates Objectified Body Consciousness Theory, Social Cognitive Theory, and Self-Determination Theory into a “Pressure-Support-Motivation” (PSM) framework. Using LPA, we identify latent profiles of exercise participants to reveal differentiated adherence mechanisms. Research questions include:

*RQ1*: Based on the six dimensions of appearance anxiety, social support, autonomous motivation, self-efficacy, body esteem, and hedonic motivation, what latent profiles of exercise participants can be identified?

*RQ2*: Do significant differences exist in exercise adherence levels across different latent profiles?

*RQ3*: How do the psychological driving mechanisms of exercise adherence vary across different latent profiles?

This study aims to make three progressive contributions: First, at the theoretical level, it integrates Objectification Theory, Social Cognitive Theory, and Self-Determination Theory to propose a “Pressure-Support-Motivation” (PSM) framework, while discussing the potential differential mechanisms of relevant variables across various populations. Second, at the methodological level, it employs Latent Profile Analysis (LPA) to identify distinct latent subgroups and utilizes Multi-Group Structural Equation Modeling (MGSEM) to examine variations in path relationships across these profiles, thereby offering a more intuitive visualization of population heterogeneity. Finally, at the practical level, it puts forward intervention principles and potential application scenarios based on population segmentation, providing a reference for future intervention studies and the validation of digital health tools.

The remainder of this paper is organized as follows: Section 2 reviews relevant theories and constructs the research framework and hypotheses; Section 3 describes the research design, sample characteristics, measures, and analytical strategy; Section 4 reports the results of the LPA and multi-group SEM; Section 5 discusses the theoretical and practical implications, limitations, and future directions; and Section 6 summarizes the core conclusions.

## 2 Theoretical framework and research hypotheses

### 2.1 Objectified body consciousness theory and appearance anxiety

Objectified Body Consciousness Theory (8) argues that in visually driven cultures, the body is constantly objectified. Individuals internalize this “external gaze,” leading to appearance anxiety—a chronic concern that one’s body fails to meet societal ideals (15). Social media further amplifies this anxiety (16).

Research on appearance anxiety and exercise shows two conflicting patterns. First, high anxiety can lead to avoidance or appearance-oriented motivation, which is linked to poorer exercise experiences and lower adherence (17, 18). Second, anxiety can also drive self-improvement through social comparison (19). This study proposes that the effect of appearance anxiety on exercise adherence is “condition-dependent”—it depends on available psychological resources. Drawing on Lazarus’s Transactional Model of Stress and Coping (20), When individuals appraise appearance anxiety as a “challenge that can be managed through effort” and possess high self-efficacy, high social support, or high autonomous motivation, appearance pressure may, under certain resource conditions, coexist with higher exercise engagement and persistence. Conversely, when individuals appraise it as a “threat” and lack coping resources, appearance pressure may be accompanied by tendencies toward exercise avoidance or dropout. Based on this analysis, this study expects that different types of individuals exist among exercise participants: some may have high appearance anxiety but also possess strong efficacy beliefs or social support, some may have lower appearance anxiety but rely on intrinsic interest to maintain exercise, and others may experience appearance anxiety but lack critical resources, leaving them on the verge of dropout. This heterogeneity is precisely the core content that latent profile analysis seeks to reveal.

### 2.2 Social cognitive theory and social support

Social Cognitive Theory (SCT; (21)) emphasizes the triadic reciprocal determinism between the person, behavior, and environment. In exercise contexts, SCT focuses on mechanisms such as self-efficacy, outcome expectations, observational learning, and environmental facilitators. As a core environmental factor, social support encompasses four functional types: emotional, informational, instrumental, and appraisal support (22). In the context of exercise adherence, social support may function through various pathways, including direct association (e.g., reducing psychological barriers, increasing social enjoyment, and accountability), enabling mechanisms (e.g., boosting self-efficacy; (3)), and stress buffering (6). However, the effects of social support are not uniform. For those with strong social needs, support may be a core driver; however, for those with high autonomy needs, excessive social attention may be perceived as controlling pressure, thereby undermining autonomous motivation (7). Through LPA, we expect to identify “Socially Dependent” and “Autonomously Driven” profiles to test the differentiated roles of social support.

### 2.3 Self-determination theory and intrinsic motivation

Self-Determination Theory (23, 24) positions exercise motivation on an autonomy continuum: from amotivation and external regulation (rewards/punishment), to introjected regulation (avoiding guilt or anxiety, e.g., appearance anxiety), identified regulation (valuing exercise benefits), integrated regulation (exercise as part of identity), and intrinsic motivation (enjoyment for its own sake). Autonomous motivation (identified, integrated, intrinsic) predicts long-term adherence, lower burnout, and higher well-being (25, 26), whereas controlled motivation relies on external pressure.

Autonomous motivation requires three basic psychological needs: autonomy, competence, and relatedness. This study focuses on three operationalized variables: (1) self-efficacy—belief in one’s ability to exercise (3), which helps persist with challenging goals (27); (2) body esteem—evaluation of one’s physical capabilities, appearance, and health (28, 29), noting it is not simply the opposite of appearance anxiety; and (3) hedonic motivation—pleasure and fun during exercise (30). According to the Affective-Reflective Theory (31), positive emotional experiences drive behavior maintenance, creating a self-reinforcing loop.

### 2.4 The “pressure-support-motivation” (PSM) framework

By integrating appearance anxiety (AA), social support (SS), autonomous motivation (AM), self-efficacy (SE), body esteem (BSE), and hedonic motivation (HM), this study constructs the “Pressure-Support-Motivation” (PSM) three-dimensional framework.

The Pressure dimension focuses on appearance anxiety, reflecting the impact of external cultural norms. The Support dimension utilizes social support as a carrier, emphasizing interpersonal resources that buffer stress and enable behavior. The Motivation dimension encompasses autonomous motivation, self-efficacy, body esteem, and hedonic motivation, focusing on the internalization of psychological drivers. It is important to clarify that the “motivation” dimension in this paper does not equate the aforementioned variables to motivation types as defined in Self-Determination Theory (SDT). Instead, they are defined as a “motivational resource system” that is more proximal to exercise persistence: autonomous motivation reflects the mode of motivational regulation and the degree of value internalization; self-efficacy reflects the belief foundation regarding competence and behavioral feasibility; body esteem reflects body-related self-concepts and value experiences; and hedonic motivation reflects immediate emotional rewards during the exercise process. Since these four variables correspond to different stages in the processes of internalization and maintenance, they serve as key indicators within the same dimension in the PSM framework for the purposes of profile identification and cross-profile comparison.

Dynamic interactions exist between these dimensions: social support can buffer the pressure of appearance anxiety and enhance self-efficacy by satisfying relatedness needs. The direction and strength of the association between appearance anxiety and exercise persistence may depend on the individual’s level of motivational resources, such as self-efficacy, body esteem, and hedonic motivation. Since variable-centered methods struggle to capture these complex interactions across specific population segments, this study employs LPA to identify latent sub-groups, thereby examining differences in exercise persistence across these subgroups and their differentiated path relationship patterns.

### 2.5 Research hypotheses

Based on the aforementioned theoretical analysis, this study proposes the following hypotheses:

Regarding the existence and characteristics of latent profiles:

• *H1*: Based on the six psychological dimensions—appearance anxiety, social support, autonomous motivation, self-efficacy, body esteem, and hedonic motivation—exercise participants can be classified into several (≥3) qualitatively distinct latent profiles.

• *H1a*: An “Intrinsically Driven” profile is expected, characterized by high autonomous motivation, high body esteem, and high hedonic motivation, with lower appearance anxiety; exercise is primarily driven by intrinsic interest and self-identity.

• *H1b*: An “Anxiety-Transformed” profile is expected, characterized by high appearance anxiety combined with high self-efficacy or social support; appearance-related pressure is effectively converted into exercise momentum through these psychological resources.

• *H1c*: A “Socially Dependent” profile is expected, with high social support as its core feature; exercise is primarily embedded in social networks, and sustained momentum is gained through co-participation with others.

• *H1d*: A “Motivation-Deficient” profile is expected, with low scores across all dimensions, lacking effective internal drivers or external support, and remaining on the periphery of exercise adherence.

Regarding the relationship between profiles and exercise adherence:

• *H2*: Significant differences exist in the level of exercise adherence across different latent profiles.

• *H2a*: The Intrinsically Driven profile exhibits the highest level of exercise adherence.

• *H2b*: The Motivation-Deficient profile exhibits the lowest level of exercise adherence.

• *H2c*: The exercise adherence levels of the Anxiety-Transformed and Socially Dependent profiles are moderate, but higher than that of the Motivation-Deficient profile.

Regarding differentiated driving mechanisms:

• *H3*: The psychological driving paths of exercise adherence differ significantly across latent profiles.

• *H3a*: In the Intrinsically Driven profile, the direct effects of body esteem and hedonic motivation on exercise adherence are the strongest.

• *H3b*: In the Anxiety-Transformed profile, self-efficacy is the most critical factor (playing a key role in pressure transformation).

• *H3c*: In the Socially Dependent profile, the direct effect of social support on exercise adherence is the most significant.

• *H3d*: In the Motivation-Deficient profile, all paths are weak, necessitating multi-dimensional collaborative intervention.

## 3 Methods

### 3.1 Research design and analytical strategy

This study employed a cross-sectional survey design. Data were collected via online questionnaires through a three-stage process: (1) Scale development and pre-testing (based on established scales with Chinese back-translation and adjustments; a pre-test with *N* = 50 ensured item clarity and reliability); (2) Formal data collection (using screening criteria on an online platform to ensure sample suitability); and (3) Data analysis (sequentially employing Latent Profile Analysis to identify participant profiles, testing for differences in adherence across profiles, and utilizing multi-group SEM to examine differentiated driving mechanisms).

### 3.2 Participants and data collection

Data were collected via the online platform “Questionnaire Star,” covering 31 provincial-level administrative regions in mainland China. Sampling combined snowball sampling with targeted invitations. To ensure sample suitability and data quality, the following inclusion criteria were set: participants must have exercised at least once a week during the past 6 months, be aged 18–45, and be able to understand and voluntarily complete the Chinese questionnaire.

Quality control measures included: (1) inclusion of attention check items (e.g., “Please select a specific option”); (2) exclusion of responses completed too quickly (<200 s); (3) exclusion of suspected patterned responses; and (4) exclusion of questionnaires with over 10% missing values. A total of 812 questionnaires were recovered, resulting in 625 valid samples after data cleaning (validity rate: 77.0%). Detailed sample characteristics are presented in Table 1. For the small amount of random missing data remaining after quality control, this study utilized Full Information Maximum Likelihood (FIML) within the Mplus analysis under the MLR estimation framework to maximize the utilization of available information and reduce bias resulting from listwise deletion.

**Table 1:** Demographic and exercise-related characteristics of the sample (*N* = 625).

Variable	Category	Frequency (*n*)	Percentage (%)
Gender	Male	301	48.2
	Female	324	51.8
Age	18–25 years	267	42.7
	26–35 years	245	39.2
	36–45 years	113	18.1
Education	High school and below	89	14.2
	Junior college/Bachelor’s degree	412	65.9
	Master’s degree and above	124	19.8
Exercise frequency	1-2 times per week	234	37.4
	3-4 times per week	267	42.7
	5 or more times per week	124	19.8
Primary exercise setting	Solo exercise	189	30.2
	Gym/Fitness center	278	44.5
	Group classes/Clubs	158	25.3
BMI category	Underweight (<18.5)	92	14.7
	Normal (18.5–24)	401	64.2
	Overweight/Obese (24)	132	21.1
Exercise duration	<3 months	145	23.2
	3–12 months	234	37.4
	>12 months	246	39.4

The sample was gender-balanced (51.8% female), with the majority of participants aged 18–35 (81.9%) and holding a junior college or bachelor’s degree (65.9%). Exercise frequency spanned a continuous range from 1-2 times per week (37.4%) to more than 5 times per week (19.8%). Exercise settings included solo exercise, gyms, and group classes.

Regarding sample size adequacy, simulation studies on LPA have demonstrated that category identification and parameter estimation are robust with a sample size exceeding 500 (32). Furthermore, according to the rule of thumb, each profile must contain a sufficient number of cases to ensure stable estimation (33). The 625 valid samples in this study are sufficient to support the estimation of 3 to 6 latent profiles.

### 3.3 Measures

All scales were adapted from established international literature and adjusted into Chinese following Brislin’s (34) back-translation procedure: two bilingual researchers translated the original English into Chinese, followed by two other bilingual researchers who back-translated the Chinese into English. Consistency between the back-translated version and the original text was compared and used to refine the items. All items were rated on a 7-point Likert scale (1 = strongly disagree, 7 = strongly agree). Construct measurements are summarized in Table 2. Control variables included demographic and exercise-related factors, such as gender, age, education level, monthly income, BMI, exercise frequency, and exercise setting (solo exercise/gym/group classes), to mitigate the potential confounding effects on main effect estimations.

**Table 2:** Summary of construct measurements.

Construct	Items	Sample Item	Source	Cronbach’s *α*
Exercise persistence (EP)	4	“I stick to my planned exercise even when I feel tired or busy.”	Corbin et al. (58)	0.889
Appearance anxiety (AA)	4	“I often worry that my body shape is not good enough.”	Cash (15)	0.912
Social support (SS)	5	“My family/friends often encourage me to keep exercising.”	Sallis et al. (59)	0.905
Autonomous motivation (AM)	6	“I exercise because I value the health benefits it provides.”	Markland and Tobin (60)	0.918
Self-efficacy (SE)	4	“I am confident that I can overcome difficulties during exercise.”	McAuley (61)	0.901
Body esteem (BSE)	5	“I feel proud of my physical capabilities.”	Franzoi and Shields (28)	0.923
Hedonic motivation (HM)	4	“The process of exercising makes me feel happy.”	Kendzierski and DeCarlo (62)	0.896

### 3.4 Ethical considerations

This research was deemed exempt from full ethical review by the lead author’s institution, as it met the criteria for minimal risk. Prior to completing the questionnaire, all participants were required to read and confirm an electronic informed consent form. The consent form explicitly stated the research objectives, data usage (for academic research only), anonymity guarantees (no personally identifiable information collected), the right to voluntary participation and withdrawal at any time, and the estimated completion time (approximately 10–15 min). Research data were stored in a de-identified format on a secure server, with access restricted to members of the research team.

### 3.5 Data analysis strategy

The data analysis was conducted across three levels: measurement validation, latent profile identification, and the testing of profile differences and mechanisms. All analyses were performed using SPSS 27.0 and Mplus 8.3.

#### 3.5.1 Assessment of measurement model and preliminary analysis

First, descriptive statistics (mean, standard deviation, skewness, and kurtosis) were calculated for all variables, and scale reliability was tested using Cronbach’s *α*. Subsequently, Confirmatory Factor Analysis (CFA) was conducted to evaluate convergent and discriminant validity. Convergent validity was assessed based on factor loadings (≥0.60) and Average Variance Extracted (AVE ≥ 0.50). Discriminant validity was examined using the Fornell–Larcker criterion, ensuring that the square root of the AVE for each construct exceeded its correlations with other constructs (35). To control for potential common method bias (CMB), Harman’s single-factor test and a comparison of single-factor CFA models were employed. Pearson correlation coefficients were also calculated to examine the initial consistency of variable relationships.

#### 3.5.2 Latent profile analysis (LPA)

For optimal profile selection, LPA was conducted using appearance anxiety (AA), social support (SS), autonomous motivation (AM), self-efficacy (SE), body esteem (BSE), and hedonic motivation (HM) as profiling indicators. Models with 1 to 6 classes were estimated sequentially using Robust Maximum Likelihood (MLR) estimation to better accommodate potential distributional deviations. Model fit was evaluated based on AIC, BIC, and aBIC (lower values indicate better fit), Entropy (≥0.70 is acceptable; ≥0.80 is ideal), the Lo–Mendell–Rubin LRT and Bootstrap LRT (*p* < 0.05 indicates the *k*-class model is superior to the *k* − 1 class model), class proportions (avoiding small classes <5%), and theoretical interpretability. After determining the optimal number of classes, mean characteristics for the six indicators were calculated for each profile and visualized to facilitate conceptual naming.

#### 3.5.3 Testing for profile differences and driving mechanisms

First, the BCH method (36) was employed to examine differences in exercise persistence (EP) across profiles. This method allows for auxiliary variable analysis while accounting for the uncertainty of profile membership, thereby reducing biases associated with “hard” classification. Mean EP scores and standard errors for each profile are reported, alongside pairwise post-hoc comparisons (Wald tests).

Second, to further characterize the profiles, Chi-square tests and ANOVAs were used to examine distributional differences in gender, age, education, monthly income, BMI, exercise frequency, and exercise setting.

**Figure 1:**
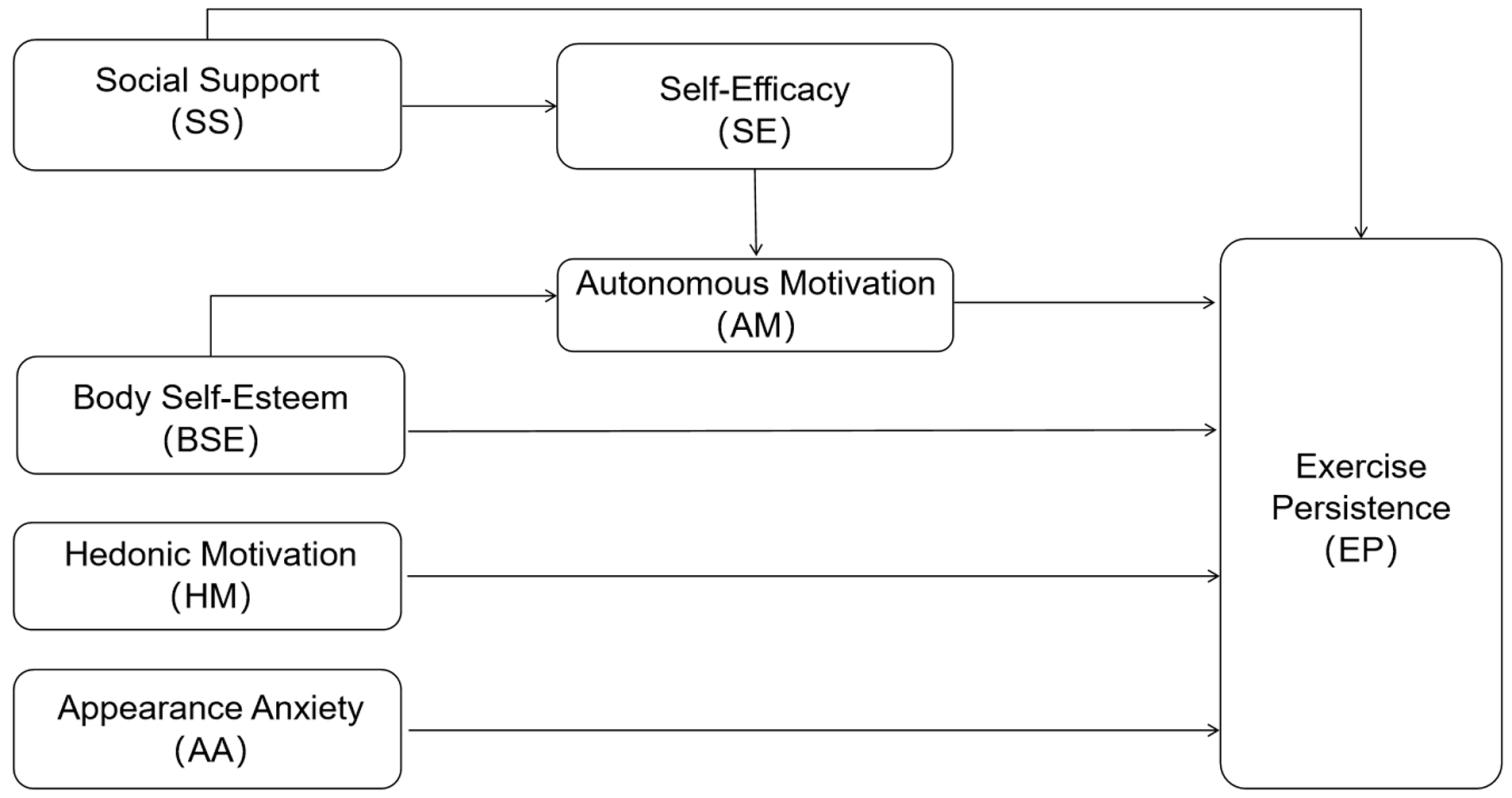
Key paths of the PSM framework.

Finally, to examine the differences in the path relationships of variables within the PSM framework across different profiles, Multi-Group Structural Equation Modeling (Multi-Group SEM) was constructed. The structural model defined key paths based on the PSM framework: SS → SE, SS → EP, SE → AM, BSE → AM, AM → EP, BSE → EP, HM → EP, AA → EP (see Figure 1). To ensure the validity of cross-group comparisons, measurement invariance (configural, weak, and strong invariance) was first tested. Following successful invariance testing, structural path differences were examined by comparing model fit between constrained and unconstrained models. Path coefficients, standard errors, and effect differences for each profile are reported to identify profile-specific core driving forces. As a robustness check, we further compared several theoretically plausible alternative structural specifications (e.g., adding an AA → AM path or removing the AA → EP path). The primary conclusions remained consistent in terms of direction and significance (see Supplementary material S1).

## 4 Results

### 4.1 Preliminary analysis

#### 4.1.1 Descriptive statistics

The descriptive statistics for all variables are presented in Table 3. The absolute values of skewness for all variables were <2, and the absolute values of kurtosis were <7, meeting the basic requirements for normality for subsequent analysis.

**Table 3:** Descriptive statistics (*N* = 625).

Variable	Mean	SD	Skewness	Kurtosis	Min	Max
Appearance anxiety (AA)	4.23	1.45	−0.12	−0.67	1.00	7.00
Social support (SS)	4.56	1.38	−0.28	−0.45	1.00	7.00
Autonomous motivation (AM)	4.89	1.32	−0.45	−0.23	1.17	7.00
Self-efficacy (SE)	4.67	1.29	−0.34	−0.31	1.00	7.00
Body esteem (BSE)	4.45	1.41	−0.21	−0.52	1.00	7.00
Hedonic motivation (HM)	4.78	1.35	−0.38	−0.28	1.00	7.00
Exercise persistence (EP)	4.52	1.48	−0.25	−0.61	1.00	7.00

Regarding the mean values, autonomous motivation (*M* = 4.89) and hedonic motivation (*M* = 4.78) were relatively high, indicating that the sample generally possesses a degree of intrinsic exercise motivation. The mean for appearance anxiety (*M* = 4.23) was at a moderately high level, reflecting the prevalence of appearance concerns among exercise participants.

#### 4.1.2 Measurement model validation

Confirmatory Factor Analysis (CFA) results indicated that the seven-factor model fit the data well (*χ*^2^/d*f* = 2.34, CFI = 0.956, TLI = 0.948, RMSEA = 0.046, SRMR = 0.038), with all indices meeting acceptable standards. The standardized factor loadings for all items were above 0.70, and the Average Variance Extracted (AVE) for each construct was >0.50, indicating good convergent validity. The square root of the AVE for each construct was greater than its correlation coefficients with other constructs (see Table 4), satisfying the Fornell-Larcker criterion and indicating good discriminant validity.

**Table 4:** Correlation matrix and discriminant validity (square root of AVE on the diagonal).

Construct	EP	AA	SS	AM	SE	BSE	HM
EP	**0.866**						
AA	0.298	**0.890**					
SS	0.567	0.234	**0.853**				
AM	0.689	0.189	0.512	**0.845**			
SE	0.612	0.167	0.534	0.598	**0.879**		
BSE	0.658	0.143	0.489	0.623	0.612	**0.876**	
HM	0.623	0.178	0.456	0.678	0.534	0.589	**0.872**

Bold values on the diagonal represent the square root of AVE; all correlation coefficients are significant at the *p* < 0.01 level.

#### 4.1.3 Common method bias test

Using Harman’s single-factor test, an unrotated exploratory factor analysis showed that the first principal component explained 36.4% of the variance, which is below the 40% threshold. Furthermore, a single-factor CFA model test showed poor fit (*χ*^2^/d*f* = 9.87, CFI = 0.68, RMSEA = 0.15), significantly inferior to the theoretical seven-factor model (Δ*χ*^2^ = 1892.45, Δd*f* = 21, *p* < 0.001), indicating that common method bias does not pose a serious threat.

### 4.2 Latent profile analysis results

#### 4.2.1 Model comparison and selection of optimal profiles

Using the six variables—appearance anxiety (AA), social support (SS), autonomous motivation (AM), self-efficacy (SE), body esteem (BSE), and hedonic motivation (HM)—as profile indicators, latent profile models with 1 to 6 classes were estimated. The model fit indices are summarized in Table 5.

**Table 5:** Comparison of latent profile models.

Profiles	AIC	BIC	aBIC	Entropy	LMR *p*	BLRT *p*	Min class %
1	12456.32	12509.45	12471.23	—	—	—	100%
2	11823.45	11907.12	11849.67	0.812	<0.001	<0.001	38.4%
3	11398.67	11513.89	11437.21	0.845	<0.001	<0.001	22.6%
4	11089.23	11235.98	11140.07	0.867	0.003	<0.001	21.4%
5	10978.45	11156.74	11041.60	0.854	0.089	<0.001	8.6%
6	10912.34	11122.17	10987.80	0.841	0.234	0.012	4.8%

From the statistical indices: AIC, BIC, and aBIC continued to decline as the number of profiles increased, but the decline significantly slowed from 4 to 5 classes (ΔBIC_4-5_ = 79 vs. ΔBIC_3-4_ = 278). Entropy reached its highest value (0.867) in the 4-class model, indicating optimal classification accuracy. The LMR test showed that the 4-class model was significantly better than the 3-class model (*p* = 0.003), while the 5-class model did not show significant improvement over the 4-class model (*p* = 0.089). Additionally, the 5-class and 6-class models contained small classes (<10%), which could compromise stability. Based on statistical indices, class size, and theoretical interpretability, the 4-class model was determined as the optimal solution. Furthermore, in Supplementary material S2, we additionally compared the profile interpretability and theoretical correspondence among the 3-class, 4-class, and 5-class solutions to further illustrate the parsimony and theoretical clarity of selecting the 4-class solution.

#### 4.2.2 Description of the four profiles

**Figure 2:**
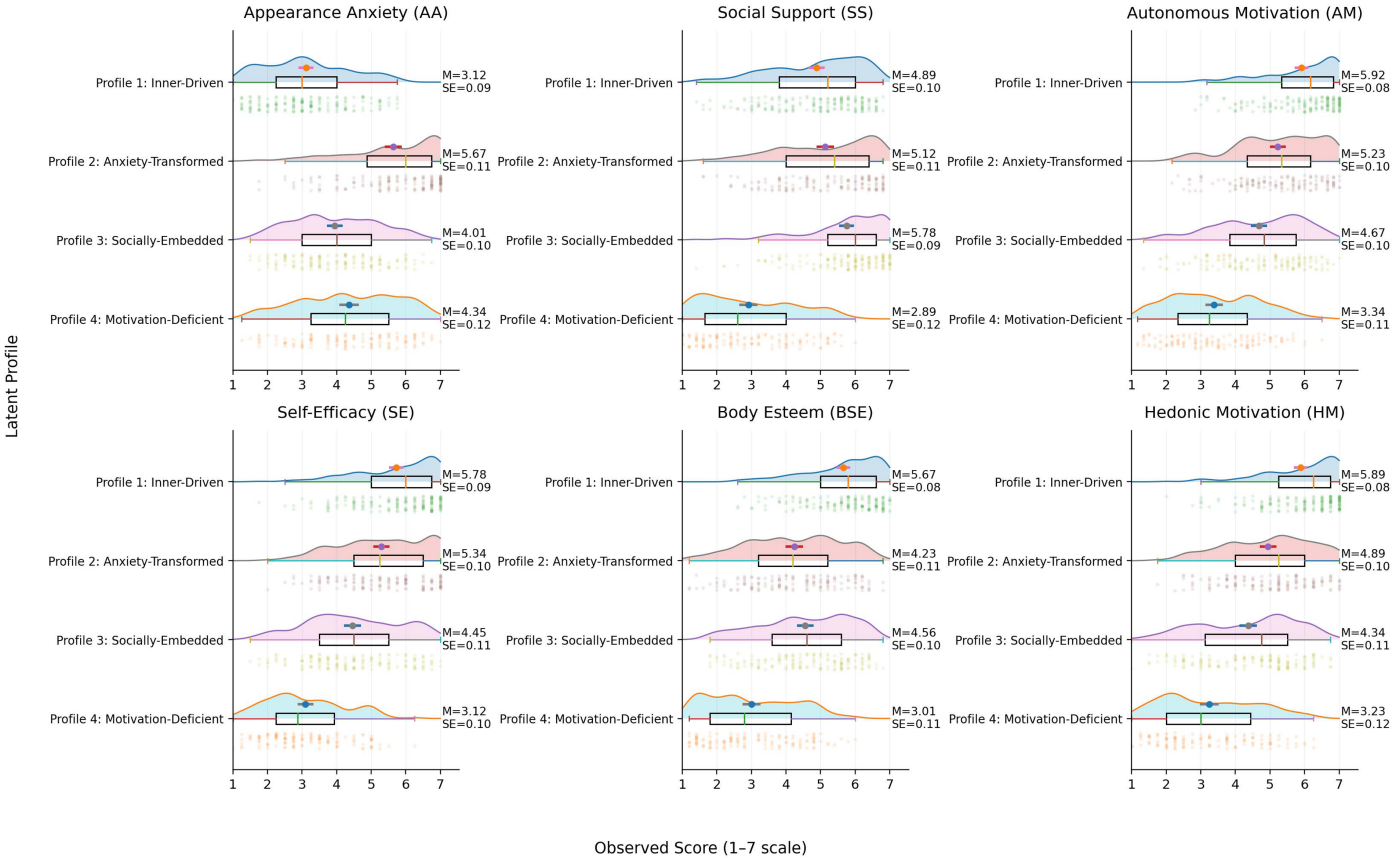
Raincloud plot of the four latent profiles across the six indicators. The raincloud plot comprehensively integrates the kernel density distribution (the “cloud”), box plot (the “box”), and raw data points/jittered points (the “rain”), and is used to simultaneously present the distributional shape, degree of dispersion, and central tendency of each profile across the indicators.

**Figure 3:**
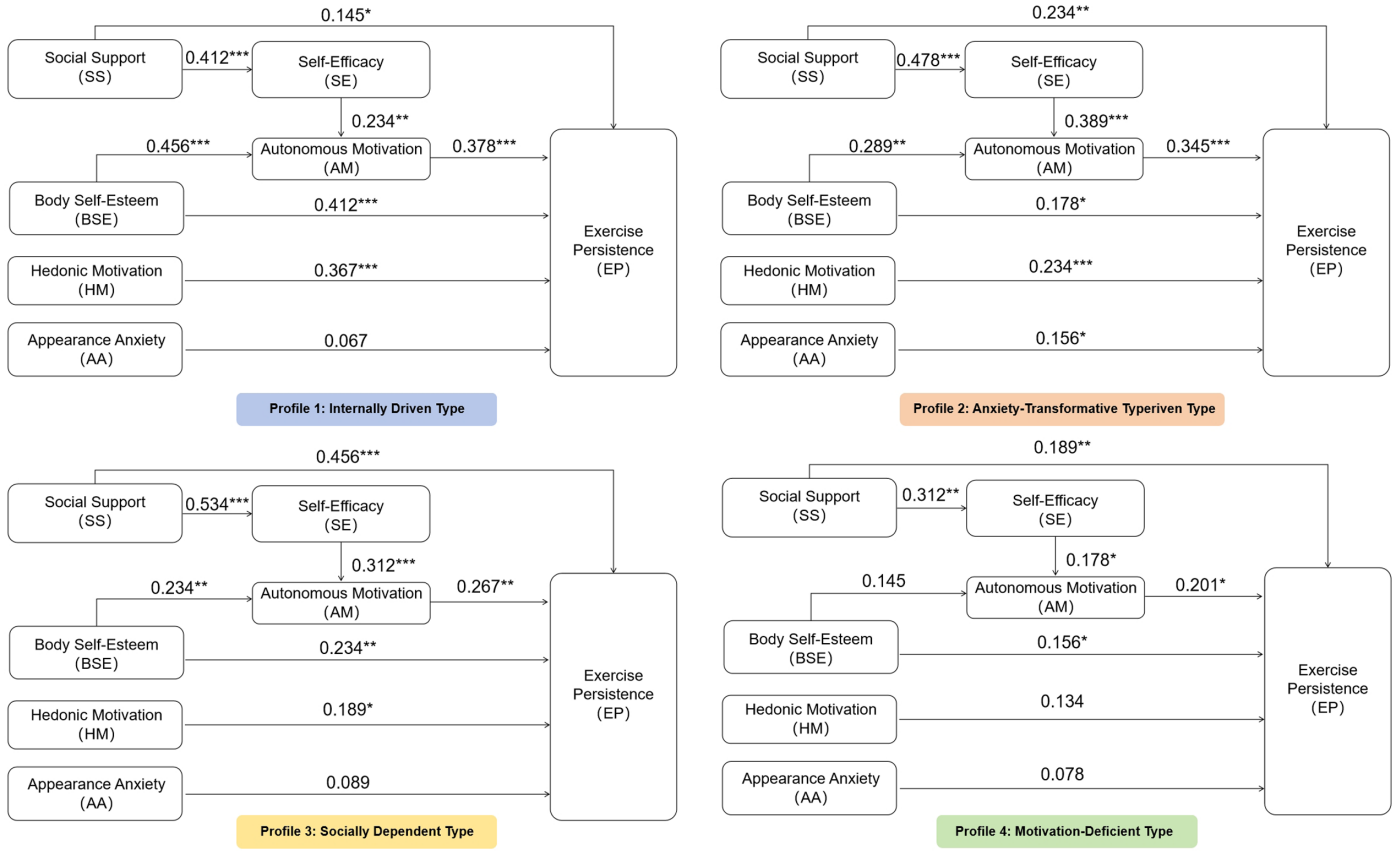
Comparison of path models across the four latent profiles.

The means and standard errors for the four profiles across the six indicators are shown in Table 6. To intuitively present the differences across these core indicators, a raincloud plot was generated (see Figure 2). Compared to mean/SE comparisons alone, raincloud plots display the overall distribution, sample dispersion, and central tendency for each variable across profiles, more comprehensively revealing heterogeneity. As shown in Figure 3, the distributional profiles for the four classes are clearly distinct: the Intrinsically Driven group shows high levels of autonomous motivation, self-efficacy, body esteem, and hedonic motivation with relatively low appearance anxiety; the Anxiety-Transformed group exhibits significantly high appearance anxiety accompanied by high self-efficacy and social support; the Socially Dependent group is characterized primarily by peak social support levels while other resources remain moderate; and the Motivation-Deficient group scores relatively low across all six indicators. Overall, Figure 3 provides a visual supplement to the mean values in Table 6, supporting the theoretical rationale for the profile naming. It is important to note that latent profiles are statistical subgroups identified based on patterns of indicator responses, used to describe heterogeneity within the sample, and do not represent fixed or immutable “types.” The naming of these profiles in this paper is intended to summarize their relatively prominent indicator characteristics (descriptive labels); terms such as “Anxiety-Transformed” are used solely for ease of communication and theoretical discussion and do not imply that individuals have necessarily undergone an observable “transformation process” over a time series.

**Table 6:** Characteristics of the four latent profiles (means and standard errors).

Variable	Profile 1 (*n* = 177, 28.3%)	Profile 2 (*n* = 151, 24.2%)	Profile 3 (*n* = 163, 26.1%)	Profile 4 (*n* = 134, 21.4%)
Appearance anxiety (AA)	3.12 (0.09)	5.67 (0.11)	4.01 (0.10)	4.34 (0.12)
Social support (SS)	4.89 (0.10)	5.12 (0.11)	5.78 (0.09)	2.89 (0.12)
Autonomous motivation (AM)	5.92 (0.08)	5.23 (0.10)	4.67 (0.10)	3.34 (0.11)
Self-efficacy (SE)	5.78 (0.09)	5.34 (0.10)	4.45 (0.11)	3.12 (0.10)
Body esteem (BSE)	5.67 (0.08)	4.23 (0.11)	4.56 (0.10)	3.01 (0.11)
Hedonic motivation (HM)	5.89 (0.08)	4.89 (0.10)	4.34 (0.11)	3.23 (0.12)

##### 4.2.2.1 Profile 1: intrinsically driven (28.3%, *n* = 177)

Lowest appearance anxiety (*M* = 3.12); highest autonomous motivation (*M* = 5.92), body esteem (*M* = 5.67), hedonic motivation (*M* = 5.89), and self-efficacy (*M* = 5.78); social support moderately high (*M* = 4.89). This profile represents an internal resource-driven maintenance model—individuals are less affected by appearance pressure, derive emotional rewards from exercise, and have integrated exercise into their self-concept. Adherence relies on autonomy and internal reinforcement, not external pressure.

##### 4.2.2.2 Profile 2: anxiety-transformed (24.2%, *n* = 151)

Highest appearance anxiety (*M* = 5.67), but also high self-efficacy (*M* = 5.34) and social support (*M* = 5.12); autonomous motivation moderately high (*M* = 5.23); body esteem relatively low (*M* = 4.23). Appearance pressure does not inevitably lead to dropout—with high efficacy and social resources, anxiety is reappraised into behavioral momentum. Low body esteem suggests transformation relies on “competence and resources” rather than stable positive body evaluation.

##### 4.2.2.3 Profile 3: socially embedded (26.1%, *n* = 163)

Highest social support (*M* = 5.78); moderate autonomous motivation (*M* = 4.67), self-efficacy (*M* = 4.45), body esteem (*M* = 4.56), and appearance anxiety (*M* = 4.01); low hedonic motivation (*M* = 4.34). Exercise is maintained through social networks. Though internal drivers are not strongest, social support provides critical external push. Reward source likely stems from social context, not subjective pleasure from exercise itself.

##### 4.2.2.4 Profile 4: motivation-deficient (21.4%, *n* = 134)

Low across nearly all dimensions: lowest social support (*M* = 2.89), autonomous motivation (*M* = 3.34), self-efficacy (*M* = 3.12), and body esteem (*M* = 3.01); appearance anxiety moderate (*M* = 4.34). The “Pressure-Resource-Motivation” chain breaks down—appearance discomfort fails to transform into efficacy, and supportive environments or internalized motives are absent. Highly prone to dropout, constituting a high-risk group.

#### 4.2.3 Demographic characteristics of profiles

To understand the composition of each group, differences in demographic and exercise-related variables were examined (see Table 7).

**Table 7:** Comparison of demographic characteristics across the four profiles.

Variable	Category	Profile 1	Profile 2	Profile 3	Profile 4	*χ*^2^/*F*	*p*
Gender	Male (%)	54.2	39.1	48.5	51.5	18.45	<0.001
	Female (%)	45.8	60.9	51.5	48.5		
Age (*M*)	—	28.9	26.4	27.8	29.3	3.89	0.009
Education	High school and below (%)	12.4	11.9	15.3	17.9	12.34	0.055
	College/Bachelor (%)	64.4	68.2	66.3	64.2		
	Master and above (%)	23.2	19.9	18.4	17.9		
Frequency	Times/Week (*M*)	4.12	3.45	3.23	2.01	45.67	<0.001
Setting	Solo (%)	38.4	27.2	18.4	36.6	56.78	<0.001
	Gym (%)	42.9	51.0	41.1	44.0		
	Group Class (%)	18.6	21.9	40.5	19.4		
Duration	<3 months (%)	8.5	19.2	22.1	49.3	67.89	<0.001
	3–12 months (%)	28.8	43.0	42.9	38.1		
	>12 months (%)	62.7	37.7	35.0	12.7		
BMI (*M*)	—	22.1	23.4	22.5	23.8	4.23	0.006

The statistical results reveal significant differences in group composition across the four profiles:

*Gender distribution*: Females are significantly higher in the Anxiety-Transformed type (60.9%), consistent with women facing greater appearance pressure (8). The Intrinsically Driven type has a slightly higher male proportion (54.2%), reflecting men’s lower appearance anxiety.

*Age characteristics*: Anxiety-Transformed type is youngest (mean 26.4 years), indicating younger groups are more susceptible to appearance pressure. Motivation-Deficient type is oldest (mean 29.3 years), possibly reflecting exhaustion after repeated failed attempts.

*Exercise frequency*: Gradient distribution across profiles—Intrinsically Driven highest (4.12 times/week), followed by Anxiety-Transformed (3.45) and Socially Embedded (3.23), with Motivation-Deficient lowest (2.01).

*Exercise settings*: Socially Embedded type participates in group classes at significantly higher rates (40.5%), validating its “socially embedded” nature. Intrinsically Driven and Motivation-Deficient types both show high solo workout rates (38.4 and 36.6%), but the former by autonomous choice, the latter due to lack of social resources.

*Exercise duration*: Most objective indicator of persistence. Among Intrinsically Driven, 62.7% have maintained exercise for over 12 months. In contrast, only 12.7% of Motivation-Deficient type reached this standard, and nearly half (49.3%) are in early stages (<3 months), facing extremely high dropout risk.

### 4.3 Relationship between profiles and exercise persistence

#### 4.3.1 Comparison of exercise persistence levels

The BCH method was employed to test the mean differences in exercise persistence (EP) across the four latent profiles. The results demonstrate that these differences are statistically significant (Wald *χ*^2^ = 267.89, d*f* = 3, *p* < 0.001). Mean EP scores are detailed in Table 8.

**Table 8:** Exercise persistence (EP) levels across profiles.

Profile	EP mean	SE	95% CI
Profile 1: intrinsically driven	5.89	0.08	[5.73, 6.05]
Profile 2: anxiety-transformed	4.78	0.10	[4.58, 4.98]
Profile 3: socially embedded	4.45	0.09	[4.27, 4.63]
Profile 4: motivation-deficient	3.12	0.11	[2.90, 3.34]

The results of the pairwise post-hoc comparisons (Wald test with Bonferroni correction) are as follows: Profile 1 > Profile 2 (Δ = 1.11, *p* < 0.001); Profile 1 > Profile 3 (Δ = 1.44, *p* < 0.001); Profile 1 > Profile 4 (Δ = 2.77, *p* < 0.001); Profile 2 > Profile 3 (Δ = 0.33, *p* = 0.012); Profile 2 > Profile 4 (Δ = 1.66, *p* < 0.001); and Profile 3 > Profile 4 (Δ = 1.33, *p* < 0.001).

The Intrinsically Driven type exhibited the highest level of exercise persistence (*M* = 5.89), which was significantly higher than the other three categories, validating H2a. Driven by intrinsic interest and self-identity, exercise for these individuals is an enjoyment rather than a task, allowing for long-term maintenance.

The Anxiety-Transformed type showed an upper-middle level of exercise persistence (*M* = 4.78). Although lower than the Intrinsically Driven type, it was significantly higher than the Socially Embedded and Motivation-Deficient types. In the profile characterized by both high self-efficacy and high social support, appearance anxiety and exercise persistence exhibit a positive association; by contrast, this association is non-significant in the other profiles. Given the cross-sectional design, this finding should be interpreted as a profile-specific association rather than a causal “transformation effect.”

The Socially Embedded type had a moderate level of exercise persistence (*M* = 4.45), significantly lower than the Anxiety-Transformed type but higher than the Motivation-Deficient type. These individuals rely on external social support to maintain exercise; should the social network change (e.g., relocation, friends quitting, or community dissolution), their persistence may face substantial challenges.

The Motivation-Deficient type exhibited the lowest level of exercise persistence (*M* = 3.12), significantly lower than the other three categories, validating H2b. Lacking effective drivers and supporting resources, these individuals exist on the periphery of exercise adherence and represent a high-risk group for early dropout.

In summary, H2 (significant differences exist in exercise persistence across different latent profiles) and its sub-hypotheses H2a, H2b, and H2c were supported.

#### 4.3.2 Robustness test after controlling for demographic variables

To ensure that the aforementioned differences were not driven by the distributional variances of demographic variables, an Analysis of Covariance (ANCOVA) was conducted, using gender, age, education level, income, BMI, and exercise frequency as covariates. The results indicated that after controlling for these variables, the effect of profile membership on exercise persistence remained significant (*F* (3, 615) = 78.45, *p* < 0.001, *η*^2^ = 0.277). The adjusted means for each profile were highly consistent with the original means, confirming the robustness of the findings.

### 4.4 Multi-group structural equation modeling: differentiated mechanisms

#### 4.4.1 Measurement invariance testing

Before performing Multi-Group SEM, we tested for measurement invariance (see Table 9).

**Table 9:** Measurement invariance test results.

Model	*χ* ^2^	d*f*	CFI	RMSEA	ΔCFI	ΔRMSEA	Result
Configural invariance	1234.56	456	0.943	0.052	—	—	Accepted
Metric invariance	1289.34	498	0.940	0.050	−0.003	−0.002	Accepted
Scalar invariance	1367.89	540	0.935	0.049	−0.005	−0.001	Accepted

Based on the recommendations of Chen (37), a more stringent invariance constraint is acceptable when ΔCFI < 0.01 and ΔRMSEA < 0.015. The results indicated that the changes from configural to weak invariance (ΔCFI = −0.003) and from weak to strong invariance (ΔCFI = −0.005) were all within the acceptable range. This demonstrates that the measurement model possesses excellent cross-group equivalence across the four profiles, thereby allowing for meaningful comparisons of structural coefficients.

#### 4.4.2 Cross-group comparison of structural models

After confirming measurement invariance, a multi-group structural equation model (MG-SEM) was estimated to test the differences across the four profiles within the PSM framework (see Table 10). First, a freely estimated model (where all paths were allowed to vary across groups) was established. Subsequently, equality constraints were applied to each path across groups one by one, and paths with significant differences were identified using the likelihood ratio test (Δ*χ*^2^) or the ΔCFI test. By modeling the paths of the four typical profiles (Intrinsically Driven, Anxiety-Transformed, Socially Embedded, and Motivation-Deficient), we observed significant differences in the mechanisms through which social support and self-efficacy drive exercise persistence (EP), as detailed in the comparison in Figure 3.

**Table 10:** Standardized path coefficients by profile.

Path	Profile 1	Profile 2	Profile 3	Profile 4	Cross-group difference
SS → SE	0.412***	0.478***	0.534***	0.312**	Δ*χ*^2^ = 12.34, *p* = 0.006
SS → EP	0.145*	0.234**	0.456***	0.189**	Δ*χ*^2^ = 18.67, *p* < 0.001
SE → AM	0.234**	0.389***	0.312***	0.178*	Δ*χ*^2^ = 14.56, *p* = 0.002
BSE → AM	0.456***	0.289**	0.234**	0.145	Δ*χ*^2^ = 16.78, *p* = 0.001
AM → EP	0.378***	0.345***	0.267**	0.201*	Δ*χ*^2^ = 8.45, *p* = 0.038
BSE → EP	0.412***	0.178*	0.234**	0.156*	Δ*χ*^2^ = 21.34, *p* < 0.001
HM → EP	0.367***	0.234**	0.189*	0.134	Δ*χ*^2^ = 15.67, *p* = 0.001
AA → EP	0.067	0.156*	0.089	0.078	Δ*χ*^2^ = 4.56, *p* = 0.207

*p < 0.05; **p < 0.01; ***p < 0.001.

#### 4.4.3 Interpretation of core driving mechanisms for each profile

##### 4.4.3.1 Profile 1: intrinsically driven

Body esteem had the strongest direct effect on exercise persistence (EP) (*β* = 0.412, *p* < 0.001) and on autonomous motivation (*β* = 0.456, *p* < 0.001). Hedonic motivation also strongly predicted EP (*β* = 0.367, *p* < 0.001). Mechanism: positive body evaluation directly promotes persistence and indirectly via autonomous motivation (“exercise is part of me”). Pleasure from exercise creates positive reinforcement. Exercise is celebration, not body change. Social support was significant but not core (*β* = 0.145). H3a supported.

##### 4.4.3.2 Profile 2: anxiety-transformed

Self-efficacy had the strongest effect on autonomous motivation (*β* = 0.389, *p* < 0.001). Appearance anxiety positively predicted EP (*β* = 0.156, *p* < 0.05)—unique to this profile. In the “Anxiety-Transformed” profile, the SE → AM path coefficient is the highest (*β* = 0.389, *p* < 0.001), and AA → EP is significantly positive only in this group among the four profiles (*β* = 0.156, *p* < 0.05). This combination presents a relational pattern consistent with the “stress reappraisal/coping resource” perspective: under the configuration of higher anxiety and higher resources, appearance pressure may be more easily associated with higher persistence, while the link between self-efficacy and autonomous motivation is stronger. Given the cross-sectional design, these results should be interpreted as differences in correlational structure rather than an observable causal transformation process. Furthermore, the SS → SE path is also relatively strong in this profile (*β* = 0.478), suggesting that social support may be accompanied by the enhancement of efficacy beliefs; however, the level of body self-esteem in this profile is relatively low (*M* = 4.23), and the BSE → EP effect is weak (*β* = 0.178), indicating that their persistence may still rely more on “competence beliefs/resource allocation” rather than a stable, positive body identity.

##### 4.4.3.3 Profile 3: socially embedded

Social support had the strongest direct effect on EP (*β* = 0.456, *p* < 0.001) and strongest indirect effect via self-efficacy (SS → SE: *β* = 0.534). This result validates the core characteristics of the “socially dependent” profile: the direct path coefficient between social support and exercise persistence is the largest in this profile, suggesting that their exercise persistence is more closely tied to social relational resources. In contrast, the path coefficients for variables related to intrinsic motivation are smaller, indicating that their persistence is more reflective of a strong association between social context and behavioral maintenance. Exercise behavior highly dependent on social networks—companionship, encouragement, community. Autonomous motivation (*β* = 0.267) and body esteem (*β* = 0.234) effects were lower than Intrinsically Driven; hedonic motivation weak (*β* = 0.189). These individuals exercise “because people are there,” not intrinsic interest. Reliance on external resources poses risk: if social support changes, persistence may falter. H3c supported.

##### 4.4.3.4 Profile 4: motivation-deficient

All path coefficients were lowest or non-significant. Body esteem → autonomous motivation non-significant (*β* = 0.145, *p* > 0.05); hedonic motivation → EP non-significant (*β* = 0.134, *p* > 0.05)—no effective internal driving system. Social support → EP small (*β* = 0.189, *p* < 0.01), drastically lower than Socially Embedded (*β* = 0.456). Low resource levels (social support *M* = 2.89) plus weak paths fail to generate momentum. This group faces dual dilemmas: low psychological resources and low conversion efficiency. H3d supported.

#### 4.4.4 Effect decomposition and mediation analysis

To further reveal the underlying mechanisms, an effect decomposition was performed (see Table 11 for the effects of SS on EP).

**Table 11:** Decomposition of effects of social support on exercise persistence.

Profile	Direct effect	Indirect (SS → SE → EP)	Indirect (SS → SE → AM → EP)	Total effect
Intrinsically driven	0.145*	0.082**	0.037*	0.264**
Anxiety-transformed	0.234**	0.095**	0.064**	0.393***
Socially embedded	0.456***	0.107**	0.045**	0.608***
Motivation-deficient	0.189**	0.062*	0.011	0.262**

SS, social support, SE, self-efficacy, AM, autonomous motivation, EP, exercise persistence. All coefficients are standardized. *p < 0.05; **p < 0.01; ***p < 0.001.

In the Socially Embedded profile, the total effect of social support was the largest (0.608), with the direct effect (0.456) being dominant; This indicates that the association between social support and exercise persistence is the most direct and has the largest coefficient for this population. In the Anxiety-Transformed profile, the indirect effect of social support was relatively more critical, exerting its influence through a chain mediation of “enhancing self-efficacy → promoting autonomous motivation.”

Conversely, in the Motivation-Deficient profile, the indirect effect pathway was obstructed (the AM → EP effect within the SS → SE → AM → EP path was weak), suggesting that even when social support is provided, it remains difficult to effectively activate their intrinsic motivational system.

### 4.5 Summary of results and hypothesis testing

To evaluate the comprehensive validity of the proposed “Pressure-Support-Motivation” (PSM) framework, the empirical findings from both the Latent Profile Analysis (LPA) and the multi-group Structural Equation Modeling (SEM) were synthesized. The prior verification of cumulative measurement invariance (configural, metric, and scalar) rigorously justified the equivalence of the measurement model across groups, ensuring that the subsequent structural path coefficients were statistically comparable. Consequently, the distinct psychological driving mechanisms and behavioral persistence patterns identified within each latent profile provided empirical evidence to validate our baseline hypotheses. To present a panoramic view of the theoretical model’s performance, the empirical results for all primary hypotheses (H1 to H3) and their respective sub-hypotheses are systematically consolidated and summarized in Table 12.

**Table 12:** Summary of research hypothesis testing.

Hypothesis	Content	Result
H1	Exercise participants can be classified into qualitatively distinct latent profiles.	Supported
H1a	Existence of an “Intrinsically Driven” type (high AM, BSE, HM; low AA).	Supported
H1b	Existence of an “Anxiety-Transformed” type (high AA, SE, SS).	Supported
H1c	Existence of a “Socially Embedded” type (high SS as core).	Supported
H1d	Existence of a “Motivation-Deficient” type (low scores across all dimensions).	Supported
H2	Significant differences in exercise persistence (EP) levels exist across profiles.	Supported
H2a	Intrinsically driven type has the highest EP level	Supported
H2b	Motivation-Deficient type has the lowest EP level	Supported
H2c	Anxiety-transformed and socially embedded types have moderate EP levels.	Supported
H3	Driving mechanisms differ significantly across profiles.	Supported
H3a	BSE and HM have the strongest effects in the Intrinsically Driven type.	Supported
H3b	SE is the most critical factor in the Anxiety-Transformed type.	Supported
H3c	SS has the most significant direct effect in the Socially Embedded type.	Supported
H3d	All paths are weak in the Motivation-Deficient type, requiring multifaceted	Supported

## 5 Discussion

### 5.1 Interpretation of key findings

Before interpreting the aforementioned results, it must be emphasized that this study employs a cross-sectional design. The structural paths presented reflect statistical associations between variables within the theoretical framework and their variations across profiles, rather than strict causal mechanisms or time-series effects. Therefore, terms such as “mechanism” and “transformation” used in the text should be understood as a theory-driven explanatory framework and evidence of association. Future research, using longitudinal tracking or intervention experiments, is required to test whether these paths constitute replicable causal processes.

#### 5.1.1 Theoretical implications of the four profiles

This study finds that exercise participants are not homogeneous but fall into four qualitatively distinct psychological profiles: Intrinsically Driven, Anxiety-Transformed, Socially Embedded, and Motivation-Deficient.

*Intrinsically driven (28.3%)*: Validates Self-Determination Theory (38). These individuals have fully internalized exercise into their identity. Their low appearance anxiety suggests positive body esteem buffers sociocultural pressures. Long-term adherence requires shifting from external goals (“changing the body”) to internal identity (“appreciating the body”).

*Anxiety-transformed (24.2%)*: Addresses the debate on appearance-driven motivation. Contrary to negative views (18), when high appearance anxiety coexists with resource characteristics such as higher self-efficacy and social support, appearance pressure and exercise persistence may exhibit a positive association; by contrast, under configurations with lower resources, this association may be attenuated or non-significant. Aligning with Transactional Stress Theory (20), self-efficacy is key: believing “I can improve through exercise” turns pressure into a challenge; low efficacy turns it into a threat and avoidance.

*Socially embedded (26.1%)*: Highlights social support’s importance (21). However, lacking strong internal drivers, exercise functions as a “social activity” rather than a “personal pursuit.” This works in stable networks but risks dropping out during life transitions (e.g., moving, job changes).

*Motivation-deficient (21.4%)*: The most concerning group. Despite no internal or external drivers, they still exercise at least weekly—likely due to external coercion, yo-yo participation, or residual habits. They are at highest risk for early dropout.

#### 5.1.2 Theoretical integration of differentiated driving mechanisms

Multi-group SEM reveals that the same psychological variables operate differently across population types (39, 40), with key implications for theory and practice.

*Body esteem*: Its direct effect on exercise persistence was strongest in the Intrinsically Driven group (*β* = 0.412) and weak in the other three. This suggests a “threshold effect”—body esteem drives adherence only when it reaches a high level. For Anxiety-Transformed and Motivation-Deficient groups with low body esteem, simply improving it is insufficient; other strategies are needed.

*Self-efficacy*: Acts as a unique “transformer” in the Anxiety-Transformed group (SE → AM path *β* = 0.389, highest across groups). This confirms that appearance anxiety requires self-efficacy to be internalized as autonomous motivation (3, 41). Without this “translation,” appearance pressure leads to frustration and avoidance.

*Social support*: Its direct effect was strongest in the Socially Embedded group (*β* = 0.456), but its indirect enabling role in the Anxiety-Transformed group (via boosting self-efficacy) is equally important. Thus, social support works differently: it directly drives behavior for Socially Embedded individuals, but serves as a psychological resource booster for Anxiety-Transformed individuals.

It should also be noted that the PSM framework does not possess sufficient explanatory power for all contexts. External situational constraints may attenuate or even interrupt these association paths, such as sudden health events, time scarcity due to work and caregiving burdens, economic pressure, sports injuries, and environments characterized by high control or stigmatizing evaluations. Under such circumstances, even if an individual possesses high self-efficacy or social support, exercise persistence may decline due to external limitations. Therefore, the results of this study are better understood as patterns of relationships between PSM variables and exercise persistence, along with their group differences, within relatively normative life contexts.

#### 5.1.3 Typological explanation of early dropout in exercise persistence

This study offers a new lens for understanding early exercise dropout (42, 43). Distribution differences show that 49.3% of the Motivation-Deficient group are in the early stage (<3 months), compared to only 8.5% of the Intrinsically Driven group. Thus, early dropout is not universal but specific to certain individuals (44). The Motivation-Deficient group struggles to cross the three-month threshold due to a lack of effective driving mechanisms—low psychological resources and low conversion efficiency (45). In contrast, the Intrinsically Driven group crossed this threshold long ago via strong internal motivation, while the Anxiety-Transformed and Socially Embedded groups (each ~20% in early stage) have viable paths of “pressure transformation” and “social support,” respectively (25, 45). Key insight for interventions: the solution is not unified incentives but first identifying an individual’s type and then providing targeted support.5.2 Theoretical Contributions.

#### 5.1.4 Methodological innovation: from variable-centered to person-centered

This study’s key methodological contribution is introducing person-centered methods to exercise persistence research (46). Traditional variable-centered methods (e.g., SEM, regression) ask “which factors influence persistence” and assume all individuals follow the same causal logic (39). In contrast, we first used Latent Profile Analysis (LPA) to identify “different types of people,” then used Multi-group SEM to examine how driving mechanisms differ across groups (47).

This shift has three benefits. First, it reveals heterogeneity masked by traditional methods: in variable-centered analysis, appearance anxiety’s overall effect might appear weak because positive effects in the Anxiety-Transformed group cancel out null effects elsewhere. Person-centered analysis shows anxiety actually drives the Anxiety-Transformed type (*β* = 0.156, *p* < 0.05) but not others (48). Second, it provides a direct basis for precision intervention—LPA outputs “population types,” which are easier to apply than abstract “variable relationships.” Fitness coaches or apps can assess user type and match strategies accordingly (49–51, 63). Third, it bridges theory and practice: while practitioners intuitively distinguish “different types of gym-goers,” this study validates and refines that intuition with rigorous methods and empirical evidence (51).

#### 5.1.5 Theoretical integration: preliminary support for and expansion of the PSM three-dimensional framework

This study proposes and provides preliminary evidence within this sample for variable relationships and cross-profile differences under the “Pressure-Support-Motivation” (PSM) three-dimensional integrated framework. Its theoretical significance lies in providing a more integrative explanatory perspective on how appearance pressure, social resources, and motivational resources relate to exercise persistence (8, 10, 23). Objectified Body Consciousness Theory (appearance pressure), Social Cognitive Theory (social support & self-efficacy), and Self-Determination Theory (intrinsic motivation) come from different traditions; this study integrates them into one framework to show their interactions (52).

Crucially, the effect of the pressure dimension (appearance anxiety) depends on the support dimension (social support) and motivation dimension (self-efficacy)—a conditional dependence that no single theory fully explains. Moreover, the PSM framework captures dynamic transformation: external pressure (appearance anxiety) can be internalized into autonomous motivation via social support buffering and self-efficacy regulation (41). The Anxiety-Transformed type empirically demonstrates this process—they begin with appearance anxiety but achieve partial motivational internalization through effective support (53).

#### 5.1.6 Contextual extension of self-determination theory

This study provides a contextualized extension of Self-Determination Theory (SDT; (10, 23)). While traditional SDT research often emphasizes the universal superiority of autonomous motivation (25, 53), our findings reveal greater complexity.

First, the paths to autonomous motivation differ by individual: for the Intrinsically Driven type, it stems from body esteem (*β* = 0.456); for the Anxiety-Transformed type, from self-efficacy (*β* = 0.389); for the Socially Embedded type, it requires social support mediation. Thus, there is no one-size-fits-all path to cultivating autonomous motivation. Second, the results of this study provide a more nuanced clue: in certain profiles characterized by higher resource conditions, appearance-related pressure and exercise persistence show a positive association, suggesting that appearance-oriented factors are not necessarily accompanied by lower persistence in all populations. However, given the cross-sectional design and the fact that this study did not directly assess long-term mental health outcomes, this finding is better understood as “preliminary supplementary evidence” for the Self-Determination Theory (SDT) motivation continuum under specific circumstances, rather than a challenge to or redefinition of existing theories (44, 53).

### 5.2 Practical implications

The core practical implication of this study is that the issue of exercise persistence should be viewed as a typological psychological mechanism challenge, rather than a problem of singular willpower or a single intervention variable (49). If the fitness industry and public health practices continue to employ a “one-size-fits-all” logic of motivational rhetoric and behavioral guidance, it may result in insufficient efficacy or even trigger resentment among key populations (51).

At the operational level, interventions should be developed around the key mechanisms of each profile:

- Intrinsically Driven type: Needs autonomy support and growth-oriented feedback to maintain identity integration (10).
- Anxiety-Transformed type: Should prioritize the construction of efficacy pathways and provide perceptible, achievable feedback on functional progress (3, 41).
- Socially Embedded type: Should reinforce the continuous supply of stable community structures and relationship networks, while gradually increasing the subjective value derived from the exercise process itself.
- Motivation-Deficient type: Requires lowering the barrier to entry, enhancing continuous companionship and accountability, and preventing the breakdown of the “resource-transformation chain” through multi-dimensional synchronous activation (42).

A corresponding profile identification-strategy matching process could be embedded into fitness apps and membership assessment systems to achieve service delivery and dynamic updates that are compatible with specific profile mechanisms (49–51, 64).

**Figure 4:**
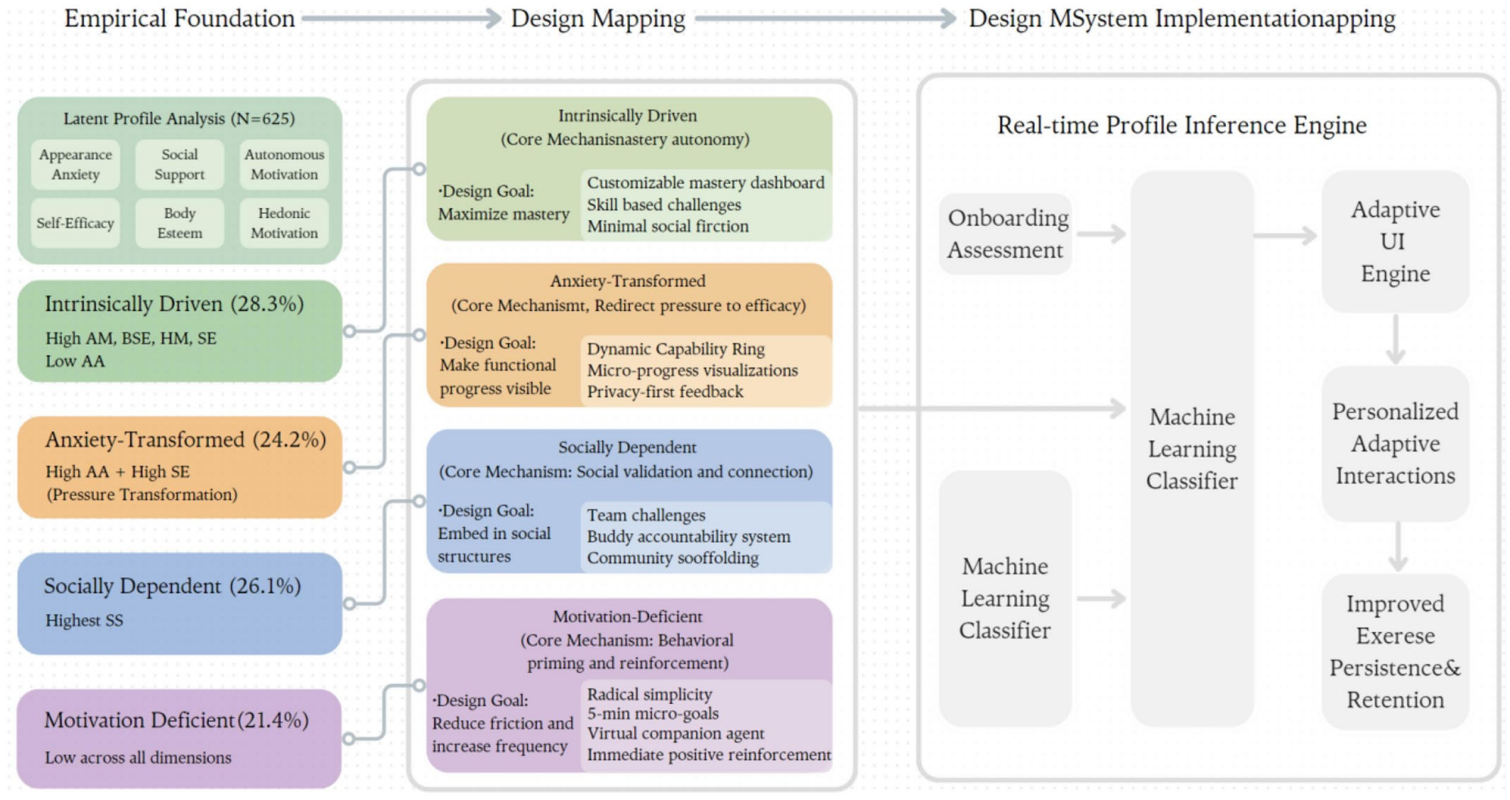
Profile-aware adaptive sports interaction framework.

To operationalize the profile-aware approach, we introduce the Profile-Aware Adaptive Sports Interaction Framework (Figure 4). This framework illustrates how the four empirically derived user profiles can be linked to distinct interaction paradigms through a real-time inference mechanism, enabling just-in-time adaptive support that aligns with each user’s dominant motivational pathways (65).

*Intrinsically Driven Users*: These users have strong inner drive and positive body perception. Avoid over-design—redundant prompts and social comparisons feel restrictive. Suggestions: (1) Allow customizable data dashboards (pace, elevation, heart rate). (2) Set skill-oriented challenges focused on athletic improvement, not body shaping. (3) Make social functions optional with private settings.

*Anxiety-Driven Users*: Motivated by body image concerns, they can turn pressure into action. Design should boost self-efficacy and prevent harmful social comparison. Suggestions: (1) Visualize small improvements to confirm workout effects. (2) Focus feedback on strength, endurance, and ability—not appearance changes. (3) Keep positive social interaction but hide or make optional appearance comparison features with strict privacy.

*Socially Dependent Users*: Social interaction is their core motivation. Simple, stable social structures are essential. Suggestions: (1) Launch team challenges to strengthen mutual supervision. (2) Add lightweight interactions to maintain social bonds without real-time participation. (3) Build community spaces for sharing and celebrating. Gradually cultivate personal interest to prevent dropout from social loss.

*Low-Motivation Users*: They lack mental energy and struggle to stick with exercise. Complex functions cause churn. Design should lower barriers and offer timely positive feedback. Suggestions: (1) Simplify onboarding with easy daily tasks, not tough goals. (2) Break workouts into short tasks with instant praise after completion. (3) Add virtual companions for gentle encouragement. Follow “progress over perfection” to build good habits first.

**Figure 5:**
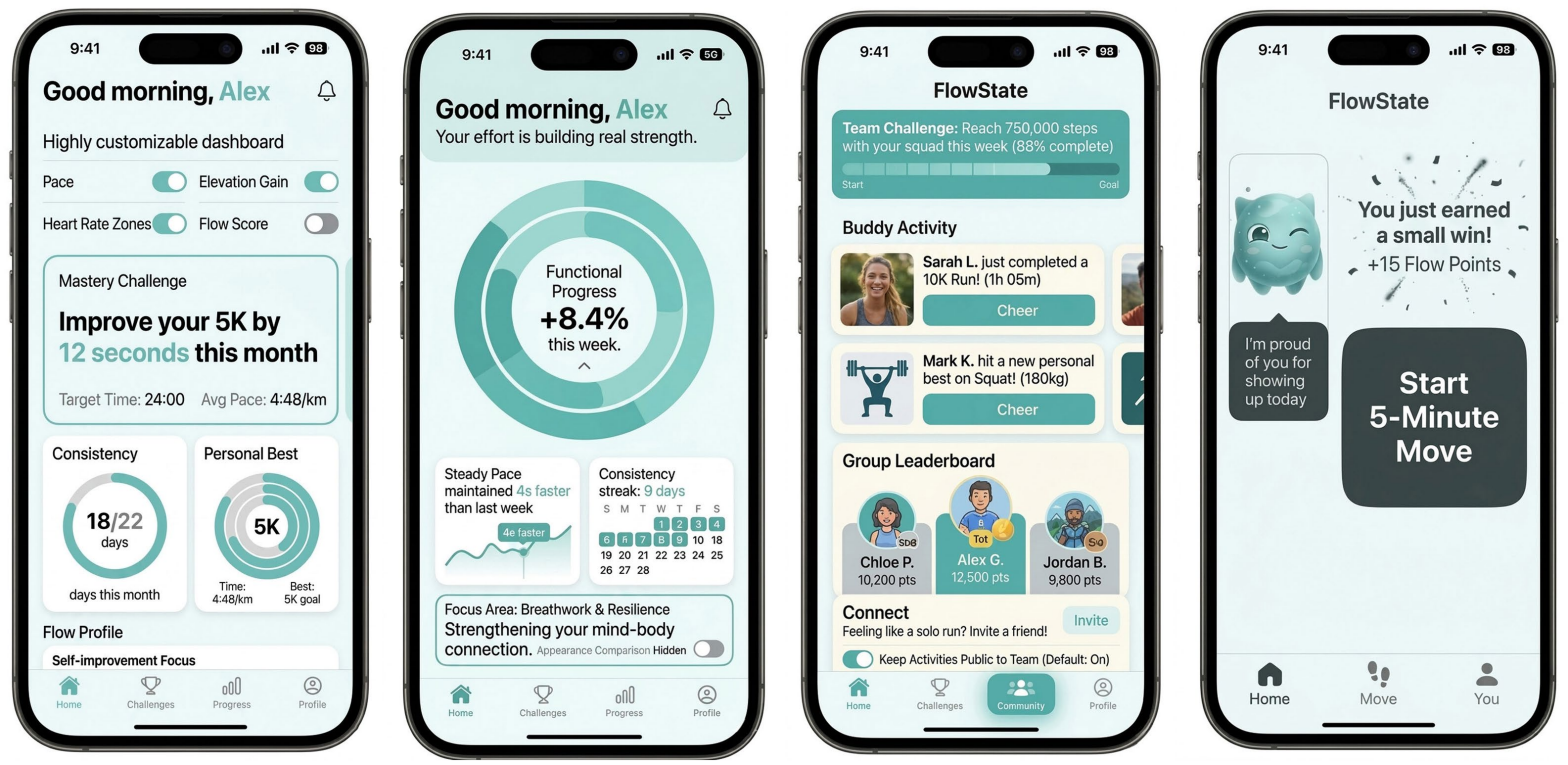
High-fidelity interface prototypes for the PeakFlow platform.

Using the adaptive sports platform PeakFlow as an example, this research designs four high-fidelity interface prototypes (Figure 5), converting psychological needs of different user groups into practical layouts, feedback modes, data visualization, and interaction logic—providing practical references for user-oriented sports product design.

### 5.3 Limitations and future directions

#### 5.3.1 Research limitations

This study has several limitations. First, the cross-sectional design limits causal inference. Although theory-driven models and multi-group SEM were used, strict causality cannot be established. For example, it is unclear whether high body esteem causes high exercise persistence or results from it—bidirectional causality may exist.

Second, the temporal stability of the four profiles remains unknown. Whether and how individuals transition between types requires longitudinal research. Key questions include whether the Anxiety-Transformed type gradually becomes Intrinsically Driven (motivational internalization) and what interventions help the Motivation-Deficient type shift.

Third, all variables were self-reported, which may introduce social desirability or recall bias, especially for exercise persistence. Future research could include objective data (e.g., wearable devices, gym check-ins) for cross-validation.

Fourth, the representativeness of the sample is limited. Although participants were recruited from all 31 provinces in mainland China, the majority were urban residents with higher levels of education. Rural, low-income, and older adult populations were underrepresented; therefore, the four profiles and their associations with exercise persistence may vary across different socioeconomic groups. Furthermore, the sample for this study consists of individuals aged 18–45 in mainland China, primarily characterized by higher education levels and urban residency. Cultural factors—such as appearance norms, the intensity of social comparison, and collectivist orientations—may influence the structural relationships among appearance anxiety, social support, and exercise behavior. Therefore, the findings of this paper should primarily be interpreted within similar cultural and demographic structures, and cross-cultural generalizability remains to be verified through replication studies in other cultural contexts.

Fifth, this study treated “exercise” holistically, without examining differences across exercise types (e.g., strength training, aerobic exercise, group sports, yoga). For instance, Socially Embedded users may prefer group sports, while Intrinsically Driven users may prefer individual activities. Future research should segment by specific exercise types.

#### 5.3.2 Future research directions

Based on the aforementioned limitations, future research can be pursued in the following directions:

- Conducting longitudinal tracking studies: A 12-month follow-up is recommended to examine profile stability and transitions. Focus on the “novice period” (0–3 months), predictors for Motivation-Deficient type transitioning, and mechanisms of Anxiety-Transformed shifting to Intrinsically Driven.
- Implementing intervention experiments: Randomized controlled trials (RCTs) can test “profile-matched interventions” against “standardized interventions.” For example, new gym members could be assigned to a “profile identification + matching strategy group” or a “standard service group” to compare 3-month persistence rates.
- Utilizing machine learning to optimize profile identification: Using larger datasets and richer features (e.g., behavioral, physiological data) to improve accuracy and real-time identification, supporting intelligent fitness systems.
- Conducting cross-cultural comparative research: Test the universality and cultural specificity of the four profiles. Focus on differences in the Socially Embedded type between collectivist (e.g., East Asia) and individualist (e.g., North America) cultures, and the impact of varying esthetic standards on the Anxiety-Transformed mechanism.
- Future research should also conduct in-depth investigations with specific populations. For example, individuals with clinical obesity may exhibit more extreme appearance anxiety and lower body esteem; older adults may prioritize social support and functional health benefits over appearance-related concerns; and adolescents, who are in a critical period of identity formation, warrant targeted studies on exercise habit development and profile differentiation.

## 6 Conclusion

Insufficient exercise persistence and early dropout signify a notable gap between exercise intention and sustained behavior (54, 55). Based on cross-sectional data from 625 adults aged 18–45 in mainland China, this study integrates Objectified Body Consciousness theory, Social Cognitive Theory, and Self-Determination Theory to propose the “Pressure-Support-Motivation” (PSM) framework. We utilized Latent Profile Analysis (LPA) and Multi-Group Structural Equation Modeling (MG-SEM) to characterize participant heterogeneity and path differences across profiles.

First, LPA identified four distinct latent profiles: Intrinsic-Driven (28.3%), Anxiety-Transformed (24.2%), Social-Dependent (26.1%), and Motivation-Deficient (21.4%), exhibiting a significant gradient in exercise persistence. Second, MG-SEM revealed distinct path differences: in the Intrinsic-Driven profile, body self-esteem and hedonic motivation showed stronger associations with persistence; in the Social-Dependent profile, social support emerged as the strongest direct predictor. Third, in the Anxiety-Transformed profile, the association between self-efficacy and autonomous motivation was strongest, and appearance anxiety exhibited a positive association with persistence—suggesting that, under high resource configurations, appearance pressure may coexist with higher persistence (a process requiring longitudinal or experimental verification). Overall, the associative pathways between psychological resources and exercise persistence vary significantly across profiles (20, 38), highlighting the need for tailored interventions that consider subgroup-specific psychological mechanisms.

This study provides person-centered evidence for understanding the heterogeneity of exercise persistence and offers testable hypotheses for future precision interventions. Future research should employ longitudinal designs to examine profile stability and transformation mechanisms (56), as well as randomized controlled trials to validate the efficacy of matching-type interventions (57). Given the cross-sectional nature and specific sample characteristics of this study, findings regarding causal mechanisms and cross-cultural generalizability require further validation in diverse populations and longitudinal contexts (3, 24).

## Data Availability

The datasets presented in this study can be found in online repositories. The names of the repository/repositories and accession number(s) can be found in the article/Supplementary material.
