# Supplementary material for: Heterogeneity among exercise participants: latent profile analysis and differential persistence pathways based on the pressure-support-motivation (PSM) framework
Source: Front Public Health. 2026 Jul 24;14:1890434. doi: 10.3389/fpubh.2026.1890434 (PMC13447197; doi:10.3389/fpubh.2026.1890434)
Supplement: Supplementary file 1 [file Table_1.DOCX]

**Supplementary Material S1**

S1. Robustness checks: alternative MG-SEM specifications

To examine whether the main conclusions were sensitive to the structural specification of the multi-group SEM (MG-SEM), we tested two theoretically plausible alternative models based on the baseline PSM structural model reported in the main manuscript.

Baseline model (PSM). The baseline MG-SEM specified the following structural paths: SS → SE, SS → EP, SE → AM, BSE → AM, AM → EP, BSE → EP, HM → EP, and AA → EP. Measurement invariance (configural, metric, and scalar) was supported in the main analysis, enabling meaningful comparisons of structural parameters across profiles.

Model A (added path). We added a direct path from appearance anxiety to autonomous motivation (AA → AM). This specification is theoretically plausible because appearance-related concerns may relate not only to persistence outcomes but also to motivational regulation processes.

Model B (trimmed path). We removed the direct path AA → EP given its limited statistical significance across profiles in the baseline model and its non-significant cross-group difference test in the main analysis.

All models were estimated using the same estimator and settings as the baseline MG-SEM (MLR; same grouping variable; same missing data handling). Model fit was evaluated using χ², CFI, TLI, RMSEA, and SRMR. Changes of ΔCFI < 0.01 and ΔRMSEA < 0.015 were treated as indicating no meaningful deterioration in fit.

As shown in Table S1-1, the fit indices of Models A and B were comparable to the baseline model, and the changes in fit indices (ΔCFI < 0.01; ΔRMSEA < 0.015) were within recommended thresholds.. Importantly, the key cross-profile patterns remained unchanged: (i) in the inner-driven profile, BSE and HM remained the strongest correlates of EP; (ii) in the socially embedded profile, SS → EP remained the strongest direct path to EP; and (iii) in the anxiety-transformed profile, the SE → AM association remained the strongest path to AM. These results suggest that the primary conclusions are robust to reasonable alternative structural specifications.

Table S1-1. Fit comparison of baseline and alternative MG-SEM models

| Model | Structural change (added/removed path) | χ² | df | CFI | TLI | RMSEA | SRMR | ΔCFI vs. baseline | ΔRMSEA vs. baseline | Notes |
| --- | --- | --- | --- | --- | --- | --- | --- | --- | --- | --- |
| Baseline (PSM) | — | 1492.36 | 560 | 0.941 | 0.936 | 0.051 | 0.043 | — | — | MLR (scaled χ²) |
| Model A | + AA → AM | 1484.90 | 556 | 0.942 | 0.937 | 0.051 | 0.042 | +0.001 | 0.000 | Added AA → AM |
| Model B | − AA → EP | 1498.77 | 564 | 0.940 | 0.935 | 0.052 | 0.044 | −0.001 | +0.001 | Removed AA → EP |

Note. χ² refers to the (scaled) model chi-square under MLR. ΔCFI and ΔRMSEA are calculated relative to the baseline model.

Table S1-2. Stability of key structural paths across baseline and alternative models

| Profile | Key path | Baseline β | Model A β | Model B β | Pattern summary |
| --- | --- | --- | --- | --- | --- |
| Inner-driven | BSE → EP | 0.412*** | 0.409*** | 0.414*** | Stable; strongest in this profile |
| Inner-driven | HM → EP | 0.367*** | 0.365*** | 0.370*** | Stable; strong positive association |
| Anxiety-transformed | SE → AM | 0.389*** | 0.385*** | 0.390*** | Stable; strongest SE→AM association |
| Socially embedded | SS → EP | 0.456*** | 0.452*** | 0.458*** | Stable; strongest SS→EP association |
| Motivation-deficient | HM → EP | 0.134 (ns) | 0.131 (ns) | 0.137 (ns) | Stable; remains weak/non-significant |

Note. *** p < .001; ns = not significant.

**Supplementary Material S2**

S2. Interpretability comparison across alternative LPA class solutions (3-, 4-, and 5-class models)

To support the selection of the four-class latent profile solution, we conducted an interpretability-based comparison of the 3-, 4-, and 5-class models in addition to the fit indices reported in the main text.

3-class solution. The 3-class model captured broad patterns that could be described as (a) a generally high-resource profile (high AM/SE/BSE/HM), (b) a profile characterized by high social support, and (c) a low-resource profile. However, this solution did not clearly separate individuals characterized by high appearance anxiety combined with relatively high efficacy/support resources from those showing more moderate or mixed resource configurations. As a result, the theoretically important “high-pressure–high-resource” configuration was partially absorbed into other classes, limiting the interpretability of pressure-related heterogeneity.

4-class solution. The 4-class model retained adequate class sizes (smallest class = 21.4%) and yielded a distinct profile characterized by high appearance anxiety alongside comparatively elevated self-efficacy and social support. This additional profile provided a clearer person-centered representation of the PSM assumption that the association pattern involving pressure may differ depending on available resources. The four profiles were therefore both conceptually coherent and readily interpretable within the PSM framework.

5-class solution. Although the information criteria continued to decrease, the LMR test was no longer significant, and a relatively small class emerged (8.6%). This additional class mainly reflected a minor deviation on a single motivation-related indicator while remaining highly similar in overall shape to an existing class. Consequently, the 5-class solution offered limited substantive interpretive gain while potentially reducing stability due to the small class size.

Conclusion. Considering statistical evidence, class size adequacy, and theoretical interpretability, the 4-class model was judged to provide the most parsimonious and theoretically meaningful representation of participant heterogeneity.In addition, the 4-class model showed the highest entropy, indicating the best classification quality among the compared solutions.
